# Economic Freedom, Education and CO_2_ Emissions: A Causality Analysis for EU Member States

**DOI:** 10.3390/ijerph19138061

**Published:** 2022-06-30

**Authors:** Gamze Sart, Yilmaz Bayar, Marina Danilina, Funda Hatice Sezgin

**Affiliations:** 1 Department of Educational Sciences, Hasan Ali Yucel Faculty of Education, İstanbul University-Cerrahpaşa, İstanbul 34500, Türkiye; gamze.sart@iuc.edu.tr; 2 Department of Public Finance, Bandirma Onyedi Eylul University, Bandirma-Balikesir 10200, Türkiye; 3 Department of Economics, Plekhanov Russian University of Economics (PRUE), 117997 Moscow, Russia; marinadanilina@yandex.ru; 4 Department of Economics, Financial University under the Government of the Russian Federation, 125167 Moscow, Russia; 5 Department of Industrial Engineering, Istanbul University-Cerrahpasa, İstanbul 34500, Türkiye; hfundasezgin@yahoo.com

**Keywords:** economic freedom, government size, international trade freedom, education, environmental sustainability, CO_2_ emissions, panel causality test

## Abstract

Environmental sustainability is one of three pillars of sustainability. However, a significant worldwide deterioration in the environment has been experienced since the Industrial Revolution, but the efforts to protect the environment date back to the 1970s. In this context, many economic and non-economic factors underlying environmental degradation have been investigated until today, but the influence of economic freedom indicators and education on the environment have been relatively less analyzed and the researchers have mainly focused on the influence of economic and institutional variables on the environment. Therefore, this paper investigates the reciprocal interplay among economic freedom indicators, education, and environment in EU member states over the 2000–2018 term by using a causality test with cross-sectional dependency and heterogeneity and taking the research gap into consideration. The causality analysis indicates that market-oriented economic structure and education can be beneficial in combatting environmental degradation.

## 1. Introduction

Environmental degradation has been one of the most serious worldwide threats that societies have faced for a long time. The atmospheric degradation, land and soil degradation, water degradation, and other types of pollution negatively affect many economic and social variables such as human health, biodiversity, climate change, disasters, and sustainable development [1,2,3]. Therefore, many goals related to the environment, including affordable and clean energy, sustainable cities and communities, clean water and sanitation, responsible consumption and production, climate action, and life on land and below water are among the 17 UN (United Nations) sustainable development goals given its wide vital implications for all living species [4].

The world has continued to experience environmental problems such as air and water pollution, climate change, disasters, and drought mainly resulting from the environmental degradation despite national, regional, and international struggles such as the Vienna Convention for the Protection of the Ozone Layer (1985), Kyoto Protocol (1997), and the Paris Agreement (2015). However, the CO_2_ emissions (metric tons per capita) have also increased to 4.5 in 2018 from 3.8 in 2000 but are substantially different between developing and developed countries [5]. Furthermore, the largest source of CO_2_ emissions is the consumption of fossil fuels, and energy-related CO_2_ emissions reached 36.3 billion tons in 2021, the highest ever level in the world [6]. The environmental indicators show that environmental degradation has continued to be a serious threat to the world.

The institutional, social, and economic factors underlying environmental degradation have been also explored by researchers in parallel with the international environmental collaborations and the studies have revealed GDP, GDP per capita, sectoral composition, urbanization, financial development, tourism, trade liberalization, FDI inflows, environmental taxes, renewable energy use, energy efficiency, circular economy, recycling, population growth, deforestation, public governance, economic and financial regulations, and the legal system as potential determinants of environmental sustainability [7,8,9,10,11,12,13,14]. In this study, the influence of economic freedom indicators and education on CO_2_ emissions is investigated by taking the research gap in the related empirical literature into consideration.

Economic freedom is defined as the ability of persons to make their own economic decisions without being exposed to intervention or limitations by government or government-supported powerful groups [15]. The main components of economic freedom are personal choice, voluntary exchange through markets, free market entry and freedom to compete in markets, and protection of property rights [16]. In sum, governments ensure an environment with the above-mentioned characteristics through institutions and regulations and provide a limited number of public goods such as infrastructure and national defense, and economic transactions are mainly implemented in the context of the free market mechanism. The countries have economic structures with different levels of economic freedom in the world. Therefore, the effect of economic freedom on economic growth and development has been widely researched in the related literature and a positive effect of market-oriented economic structures on economic growth and development has been mainly revealed [17,18,19,20].

However, economic freedom can influence environmental quality through diverse channels. One view suggests that a larger government size probably decreases the environmental quality through inefficient operations by government and state-owned enterprises [21,22]. Another view suggests that governments are crucial actors in the design and application of environmental regulations, clean energy, and green products and, in turn, can positively influence the environmental quality [23]. Furthermore, higher economic freedom can decrease the environment quality due to the use of more energy and natural resources, considering its positive growth effect depends on the economic development levels of the countries in the context of the EKC (Environmental Kuznets Curve) hypothesis [24]. However, many efficient resources can also be used to control the environment in countries with higher economic freedom and, in turn, developments in energy-efficient technologies and renewable energy production can raise the environmental quality [20]. Lastly, countries with higher economic freedom levels can efficiently use market-based instruments such as environmental taxes and tradable permit systems to improve the environmental quality. As a consequence, the impact of economic freedom and government size on the environment varies based on which channels are dominant.

The freedom of international trade or trade openness can also affect the environmental quality through diverse channels. First, the positive growth effect of trade openness can influence the environmental quality depending on the economic development levels of the countries in the context of the EKC hypothesis [25,26,27]. Secondly, trade openness can improve the environmental quality by easing the countries to reach cleaner and energy-efficient technologies and renewable energy [22,28]. Thirdly, trade liberalization can change the industry composition and influence the environmental quality based on their factor endowments [25]. As a result, the impact of international trade freedom on the environment changes depending on which channels are dominant. Economic freedom, government size, and freedom of international trade can affect the environment through various channels, but a deteriorated environment can also direct the countries to improve the environment through market-based solutions. Therefore, a mutual interplay between economic freedom indicators and the environment can exist in theoretical terms.

Education can contribute to environmental quality by raising environmental awareness and developing green and energy-efficient technologies and renewable energy production [29,30]. However, education can negatively influence the environmental quality by fostering economic growth through human capital, innovation, competitiveness, and entrepreneurship depending on the economic development levels of the countries in the context of the EKC hypothesis. On the other hand, governments can use education as an instrument in combatting environmental degradation. Similarly, a mutual interplay between education and the environment can exist theoretically.

Consequentially, economic freedom and its components of government size and freedom to international freedom, and education are expected to affect environmental sustainability. This article investigates the causal interplay among economic freedom indicators, education, and the environment proxied by CO_2_ emissions in a sample of the European Union (EU) economies. The EU environment policy goes back to the European Council meeting of 1972 in Paris, which suggested a common environment policy for the EU, and then The Single European Act of 1987 included an ‘Environment Title’, the first legal foundation for the EU common environment policy. The Treaty of Maastricht (1993) also accepted the environment as a formal EU policy area and combatting climate change became a specific goal of the EU with the Treaty of Lisbon (2009) [31]. The precaution, prevention, and rectification of pollution at the source and polluter fines are the main principles of the EU environment policy [31]. The EU targets a 55% decline in greenhouse gas emissions by 2030 compared to 1990 levels and to become the first climate-neutral continent by 2050 [32]. The figures indicated that greenhouse gas emissions decreased by 31% in the EU between 1990–2020 [33] and verified the success of the EU environment policies.

The researchers have analyzed the effect of many variables including GDP, GDP per capita, sectoral composition, urbanization, financial development, tourism, trade liberalization, FDI inflows, environmental taxes, renewable energy use, energy efficiency, circular economy, recycling, population growth, deforestation, public governance, economic and financial regulations, and legal system on the environment [7,8,9,10,11,12,13,14], but education and economic systems of a country are also very important for the environment because both education and economic freedom have an effect on the environment directly or indirectly through these variables. However, the interaction among education, economic freedom, and the environment has not been sufficiently investigated yet. Therefore, this research purposes to contribute to environmental economics by investigating the effect of economic freedom, its components, and education on the environment. Another contribution of the paper is to analyze the causal interaction among education, economic freedom, and CO_2_ emissions at a country level because the limited literature about the interplay of economic freedom, education, and environment has mainly utilized the regression approach and therefore reached one coefficient for all countries in the panel. Furthermore, the researchers have mainly analyzed the effect of institutional, social, and economic variables on the environment and have not investigated the impact of the environment on economic, social, and institutional variables. Therefore, another novelty of the article is to analyze the reciprocal interplay between education, economic freedom indicators, and CO_2_ emissions. The next section of the research reviews and sums up the literature and variables and study methods are described in Section 3. The causality analysis is performed and its findings are discussed in Section 4. The article eventuates in the Conclusion section.

## 2. Literature Review

Environmental sustainability has come into prominence in the world as of the 1970s and, in turn, precautions for environmental protection have begun to be seriously discussed at the national and international levels. In this context, researchers have also analyzed the relationship between a great number of institutional, economic, and social variables and the environment and many of them have been suggested as the determinants of environmental sustainability. In this study, we analyze the reciprocal interplay between economic freedom indicators, education, and CO_2_ emissions through the research gap in the related literature.

The limited empirical literature on the nexus between economic freedom and the environment has generally utilized the regression approach and found mixed findings. Odugbesan et al. [14], Cheon et al. [34], Adesina and Mwamba [35], Bjørnskov [36], and Jain and Kaur [37] discovered a negative influence of economic freedom indicators on CO_2_ emissions, but Wood and Herzog [38], Rapsikevicius et al. [39], Chen [24], and Alola et al. [40] have revealed mixed findings on the influence of economic freedom indicators on CO_2_ emissions.

Odugbesan et al. [14] analyzed the determinants of the green economy in Turkey for the period of 1996–2019 through time series analysis and revealed that economic freedom is a significant determinant of carbon productivity in the short and long run. On the other hand, Cheon et al. [34] investigated the effect of economic freedom on CO_2_ emissions in 111 countries over the 2005–2013 period through a regression approach and revealed a negative influence of the economic freedom index on CO_2_ emissions. Adesina and Mwamba [35] analyzed the effect of economic freedom indicators (business freedom index, fiscal freedom index, trade freedom index, and freedom from corruption index) on CO_2_ emissions in 24 African economies over the 1995–2013 term via a dynamic regression approach and discovered a negative effect of economic freedom indicators on CO_2_ emissions.

Bjørnskov [36] investigated the influence of economic freedom on greenhouse gas emissions in 105 economies with five-year periods for the 1975–2015 period via a regression approach and uncovered that economic freedom decreased the greenhouse gas emissions, but slid the maximum point of the EKC curve to the left. On the other hand, Jain and Kaur [37] examined the effect of economic freedom indicators of government size and law and order on CO_2_ emissions in Asian economies over the 1981–2016 period through a dynamic regression approach and pointed out that a higher government size index (or more market-oriented economies) and higher law and order decreased CO_2_ emissions.

Wood and Herzog [38] investigated the interaction between economic freedom indicators and air quality represented by CO_2_ emissions and concentrations of fine particulate matter in 105 countries for the 2000–2010 period and found that a 1% increase in the economic freedom index decreased concentrations of fine particulate matter by 7.15% in the long-term, but did not find a significant interaction between economic freedom and CO_2_ emissions. On the other hand, Rapsikevicius et al. [39] analyzed the effect of 10 economic freedom indicators on environmental performance in 23 European economies over 2005–2018 duration by means of correlation analysis, index calculation, clustering, trend analysis and reached mixed findings between economic freedom indicators and environmental performance indicators.

Chen [24] investigated the effect of government size in BRICS economies for the 1990–2018 period by means of the ARDL approach and discovered a positive effect of government size on CO_2_ emissions in Brazil, China, India, and South Africa in short and long-term, but a negative long-term effect of government size on CO_2_ emissions in Russia. Alola et al. [40] also analyzed the effect of economic freedom indicators of law and order, freedom to trade internationally, regulation, and sound money on the ecological footprint in G-20 countries for the period of 2000–2016 through a dynamic regression approach and found that economic freedom indicators negatively influenced the environmental quality of the countries under consideration.

The empirical studies on the nexus between education and CO_2_ emissions have stayed inconclusive in line with the theoretical considerations. Duarte et al. [41], Uddin [29], Wang et al. [42], Li and Ullah [43] revealed a decreasing effect of education on CO_2_ emissions, but Li and Zhou [44] and Zafar et al. [45] discovered an increasing effect of education on CO_2_ emissions. Furthermore, Khan [46] and Cui et al. [47] reached findings incompatible with the EKC hypothesis.

Duarte et al. [41] explored the factors underlying CO_2_ emissions in Spanish households and discovered that higher education levels decreased the CO_2_ emissions. On the other hand, Uddin [29] investigated the effect of education on CO_2_ emissions in Bangladesh for the 1974–2010 period with cointegration analysis and pointed out that education decreased the CO_2_ emissions by increasing the environmental awareness. Wang et al. [42] also analyzed the influence of education on environmental attitudes in China employing data from the Chinese General Social Survey of 2010 version and revealed that education fostered pro-environmental behaviors, but the impact of education on environmental behaviors was heterogeneous among the persons. Li and Ullah [43] analyzed the influence of education on CO_2_ emissions in BRICS economies for the 1991–2019 period via the NARDL approach and unveiled that improvements in education decreased the CO_2_ emissions.

Li and Zhou [44] explored the relationship among demographic characteristics, higher education, and CO_2_ emissions in China via fully modified ordinary least squares and disclosed a positive effect of higher education on CO_2_ emissions in East China. Zafar et al. [45] also explored the factors underlying CO_2_ emissions in 22 top remittance-receiving countries for 1986–2017 via Westerlund and Edgerton cointegration test and discovered a positive influence of education on CO_2_ emissions.

Khan [46] analyzed the effect of education on CO_2_ emissions in 122 countries for 1980–2014 via threshold regulation and discovered an inverted U-shaped interaction between education and CO_2_ emission. Cui et al. [47] also researched the influence of education on CO_2_ emissions in China for 1991–2019 via the ARDL approach and disclosed an inverted U-shaped interaction between educational indicators and CO_2_ emissions. Balaguer and Cantavella [48] analyzed the relationship between education and the environment in Australia for the 1950–2014 period and uncovered the findings in favor of the EKC hypothesis. Subramaniam and Masron [49] analyzed the effect of education on the environment in 22 developing countries for 1990–2016 via the ARDL approach and disclosed that the positive effect of poverty on environmental degradation can decrease after a certain threshold of educational attainment. Furthermore, some researchers have investigated the relationship between human capital highly dependent on education and CO_2_ emissions and found mixed findings [50,51,52,53,54,55] similar to the literature.

Based on the presented literature research, the research hypotheses of the study are:

**Hypothesis** **1.** *There is significant causality between economic freedom and CO_2_ emissions.*

**Hypothesis** **2.** *There is significant causality between government size and CO_2_ emissions.*

**Hypothesis** **3.** *There is significant causality between international trade freedom and CO_2_ emissions.*

**Hypothesis** **4.** *There is significant causality between education and CO_2_ emissions.*

## 3. Data and Methodology

The causal interplay between economic freedom, government size, freedom to international trade, education, and environment is analyzed in a sample of the EU member states over the 2000–2018 term. The environment (CO) is substituted by CO_2_ emissions (metric tons per capita). On the other hand, economic freedom is proxied by the economic freedom index (EF), government size (GOV), and international trade freedom (ITF). The government size (GOV) shows the degree of the political process in the allocation of resources, goods, and services. The government size index gets valued between 0 and 10 and higher scores reflect that a country depends on personal choice and markets rather than political decision-making processes. The index of international trade freedom (ITF) is an indicator of trade liberalization and gets valued between 0 and 10 and higher scores reflect that a country has relatively lower restrictions on international trade (see Fraser Institute [56]) for more information about the methodology of economic freedom and its subcomponents). Education (EDU) is substituted by tertiary school enrollment (rate of total tertiary enrollment to the number of individuals officially corresponding to the tertiary education level). The data on CO_2_ emissions and education are procured from World Bank [5,57] and the indicators of economic freedom are procured from Fraser Institute [56]. All series are yearly and their period is 2000–2018 because the yearly indicators of economic freedom are available as of 2000 and the CO_2_ emissions end in 2018. The econometric tests are conducted by means of EViews 11.0, and Stata 16.0. The sample includes 24 EU members (Belgium, Bulgaria, Croatia, Cyprus, Czechia, Denmark, Estonia, Finland, France, Greece, Hungary, Ireland, Italy, Latvia, Lithuania, Malta, Netherlands, Poland, Portugal, Romania, Slovakia, Slovenia, Spain, and Sweden) except Austria, Germany, and Luxembourg due to absence of education data.

The descriptive characteristics of the series are shown in Table 1. The mean of CO_2_ as metric tons per capita is 7.6895 and the mean of tertiary school enrollment is 64.1684% in the sample, but both variables substantially vary among the countries. However, the mean economic freedom index, government size index, and index of freedom to international trade are, respectively, 7.6141, 5.9805, and 8.3420, and the economic freedom index and its components are relatively more stable among the countries.

The causal interplay between indicators of economic freedom, education, and CO_2_ emissions is investigated with Emirmahmutoglu and Kose [58] causality test. The test improves the LA-VAR (lag-augmented vector autoregression) approach of Toda and Yamamoto depending upon meta-analysis for causality analysis between two variables in heterogeneous panel datasets. This test regards the level VAR model with $L_{i}+dmax_{i}$ [58]:

(1) $$y_{i,t}=\delta_{1i}+\overset{L_{i}+dmax_{i}}{\underset{l=1}{\sum }}\alpha_{1i,l}y_{i,t-l}+\overset{L_{i}+dmax_{i}}{\underset{l=1}{\sum }}\beta_{1i,l}x_{i,t-l}+\varepsilon_{1i,t}$$

(2) $$x_{i,t}=\delta_{2i}+\overset{L_{i}+dmax_{i}}{\underset{l=1}{\sum }}\alpha_{2i,l}y_{i,t-l}+\overset{L_{i}+dmax_{i}}{\underset{l=1}{\sum }}\beta_{2i,l}x_{i,t-l}+\varepsilon_{2i,t}$$

*i* (*i* = 1,2,…,*N*) indicates the cross-sections, *t* (*t* = 1,2,…,T) indicates the time dimension of the series. $L_{i}$ is the lag structure and may vary among the cross-sections, $dmax_{i}$ is maximum integration level and $\varepsilon_{1i,t}$ and $\varepsilon_{2i,t}$ are error terms.

The null hypothesis for Equation (1) suggests that $x_{i,t}$ is not Granger cause of $y_{i,t}$ for all cross-sections. Similarly, the null hypothesis for Equation (2) suggests that $y_{i,t}$ is not Granger cause of $x_{i,t}$ for all cross-sections.

## 4. Results and Discussion

In the empirical analysis, cross-sectional dependency and heterogeneity properties of the series are firstly investigated by means of LM, LM CD, LM_adj._, and delta tests, and the test consequences are reported in Table 2. The probability values of cross-sectional dependence tests (LM, LM CD, and LM_adj_.) are lower than 1%. Therefore, the null hypothesis (absence of cross-sectional dependence) is denied and the subsistence of cross-sectional dependence between series is found. The findings of cross-sectional dependence tests indicate that indicators of economic freedom, education, and CO_2_ emissions in one of the EU countries may influence the other EU member countries thanks to the EU and highly integrated world. On the other hand, the probability values of homogeneity tests are also found to be lower than 1%. As a consequence, the null hypothesis (absence of heterogeneity) is denied and the subsistence of heterogeneity is revealed. In other words, panel estimates include country-specific heterogeneity.

The maximum integration level of the variables should be determined before the application of the causality test. Therefore, the integration level of the series is analyzed using the Cross-Sectionally augmented Im–Pesaran–Shin (IPS) [59] (CIPS) test developed by Pesaran [60] thanks to cross-sectional dependency between the five series. The test findings are shown in Table 3 and, in turn, the null hypothesis (presence of a unit root) is accepted for the level values of the variables because test statistics are revealed to be lower than the critical values presented in Pesaran [60]. However, the null hypothesis is denied for the first differences of the series. In conclusion, test findings indicate that CO, EF, GOV, ITF, and EDU are I (1) and the maximum integration level is specified as 1.

In the causality analysis, the causal interplay between economic freedom (EF) and CO_2_ emissions (CO) is firstly examined with the bootstrap causality method thanks to the subsistence of cross-sectional dependence and heterogeneity, and the results are reported in Table 4. The causality findings reveal a mutual interplay between economic freedom and CO_2_ emissions at the panel level and in Denmark and Portugal but a one-way causal effect from EF to CO in Czechia, Ireland, Romania, and Spain and a one-way causal effect from CO to EF in Cyprus, Finland, and Greece.

A reciprocal interaction between economic freedom and CO_2_ emissions is theoretically expected and the panel-level causality analysis uncovers a bidirectional causality between economic freedom and CO_2_ emissions in line with the theoretical expectations. However, country-level causality analysis reveals a mutual interplay between economic freedom and CO_2_ emissions in Denmark and Portugal, but one-way causal effect from economic freedom to CO_2_ emissions in Czechia, Ireland, Romania, and Spain and a one-way causal effect from CO_2_ emissions to economic freedom in Cyprus, Finland, and Greece. Economic freedom can affect the environment through relatively more efficient allocation of resources, market-based environmental instruments, and economic growth channels. On the other side, the significant environmental degradation also can lead the countries to make the market-oriented structural reforms. The differences in country-level causality analysis can result from country-specific characteristics such as current economic development level and economic structures. In the related limited empirical literature, Rapsikevicius et al. [39] have reached mixed findings on the interaction between economic freedom indicators and environmental performance indicators for 23 European economies. On the other hand, Odugbesan et al. [14] discovered a positive effect of economic freedom on carbon productivity for Turkey. Cheon et al. [34], Adesina and Mwamba [35], Bjørnskov [36], Jain and Kaur [37], and Wood and Herzog [38] revealed a negative influence of economic freedom on CO_2_ emissions for different country groups through panel regression analysis. In this context, our findings are in accord with the related empirical literature.

The causal interplay between government size (GOV) and CO_2_ emissions (CO) is examined with the bootstrap causality method and the results are reported in Table 5. The causality findings reveal a mutual interplay between two variables at the panel level and in Italy, Malta, and Spain, but a one-way causal effect from GOV to CO in Cyprus, Czechia, Ireland, Slovenia, and Sweden and a one-way causal effect from CO to GOV in Bulgaria, Estonia, and Greece.

The two-way interaction between government size and CO_2_ emissions by causality analysis is revealed to be compatible with theoretical expectations because the government is a dominant factor in the design, application, and control of environmental and economic policies and encouraging clean energy and green products. On the other hand, governments can change the structure of the government sector to combat environmental degradation. However, country-level causality analysis reveals a mutual interplay between government size and CO_2_ emissions in Italy, Malta, and Spain, but unidirectional causality from government size to CO_2_ emissions in Cyprus, Czechia, Ireland, Slovenia, and Sweden and a unilateral causality from CO_2_ emissions to government size in Bulgaria, Estonia, and Greece. The differences in the findings of the country-level causality analysis can result from government sector characteristics and national regulatory framework. In the related empirical literature, a few scholars have analyzed the interaction between government size and CO_2_ emissions. In this context, Jain and Kaur [37] reveal that higher government size (or more market-oriented economies) decrease the CO_2_ emissions in Asian economies, but Chen [24] discovers a positive effect of government size on CO_2_ emissions in Brazil, China, India, and South Africa in the short and long term and a negative long-term effect of government size on CO_2_ emissions in Russia. So, the empirical findings suggest that government size matters for CO_2_ emissions for different countries, and our findings are mainly in line with the limited empirical findings.

The causal interplay between freedom to international trade (ITF) and CO_2_ emissions (CO) is examined with the bootstrap causality method and the results are reported in Table 6. The causality findings reveal a one-way causal effect from ITF to CO at the panel level, and in Croatia, Hungary, Italy, and Sweden, a bilateral interplay between two series in France and Spain, and a one-way causal effect from CO to ITF only in Denmark.

The unidirectional causality from international trade freedom to CO_2_ emissions by panel-level causality analysis is in line with the theoretical expectations because trade freedom or trade liberalization can influence the environment by enhancing the economic growth, changing the industry composition, and making it easy for the countries to reach cleaner or energy-efficient technologies and renewable energy. Furthermore, the country-level analysis reveals a bidirectional causality between international trade freedom and CO_2_ emissions in France and Spain; a unidirectional causality from international trade freedom to CO_2_ emissions in Croatia, Hungary, Italy, and Sweden; and a unilateral causality from CO_2_ emissions to international trade freedom. In the related empirical literature, Adesina and Mwamba [35] revealed a negative effect of the trade freedom index on CO_2_ emissions in 24 African economies. Alola et al. [40] reached similar findings for G-20 countries. Therefore, our findings and the related empirical literature point out that international trade freedom is a significant determinant of CO_2_ emissions for developing and developed countries.

The causal interplay between education (EDU) and CO_2_ emissions (CO) is examined with the bootstrap causality method and the results are reported in Table 7. The causality findings revealed a mutual interplay between two series at the panel level and in Croatia, and one-way causal effect from EDU to CO in Italy, Netherlands, Poland, Portugal, Spain, and Sweden and a one-way causal effect from CO to EDU only in Belgium, Denmark, Ireland, and Latvia.

Education is theoretically expected to influence the environmental quality by enhancing the economic growth and development, raising the environmental awareness, and developing green and energy-efficient technologies and renewable energy production. Therefore, the findings of panel-level and country causality analyses mainly support the theoretical expectations. Furthermore, the related empirical literature has generally found a significant influence of education on the environment [29,41,42,43,44,45,46,47]. Consequently, education is one of the crucial instruments to improve environmental quality.

## 5. Conclusions

Environmental degradation is one of the critical problems of the globalized world. The implications of a degraded environment such as climate change, air and water pollution, drought, and loss of biodiversity have been experienced in every part of the world and have become a serious threat to the environment and economic sustainability. Therefore, economic and non-economic determinants of environmental degradation have been intensely explored by researchers. However, the impact of economic freedom and education, which can affect all other significant determinants of environmental degradation, on the environment has not been explored sufficiently. Furthermore, the studies analyzing the interaction between economic freedom and the environment have generally conducted one-way panel analyses, in other words, studied the impact of economic freedom indicators on the environment, and disregarded the country-specific characteristics. Therefore, this study investigates the reciprocal interaction between economic freedom indicators, education, and CO_2_ emissions by means of the bootstrap Granger causality test with cross-sectional dependency and heterogeneity in a sample of the EU members that have been successful in improving environmental sustainability, to make a contribution to the related literature.

Both panel and country-level causality analyses point out that economic freedom, government size, international trade freedom, and education are significant determinants of environmental degradation proxied by CO_2_ emissions, although country-level findings partially differ depending on country-specific characteristics in line with the theoretical expectations. Therefore, reforms toward market-oriented economic structures and education can be used effectively to combat environmental degradation by using market-based environmental instruments, raising environmental awareness, and developing green or energy-efficient technologies. Future studies can be conducted on the interaction between economic freedom and the development of green or energy-efficient technologies.

## Figures and Tables

**Table 1:** Descriptive statistics of the series.

Characteristics	*N*	Observations	CO	EF	GOV	ITF	EDU
Mean	27	513	7.6895	7.6141	5.9805	8.3420	64.1684
Maximum	27	513	25.6687	8.3600	7.8333	9.5009	142.8520
Minimum	27	513	2.9271	5.4400	4.2149	6.1060	19.5623
Std.Dev.	27	513	3.5814	0.4254	0.8549	0.5638	17.5713

**Table 2:** Results of cross-sectional dependence and heterogeneity tests.

Test	Test Statistic	*p* Value
LM	691.3	0.0000
LM CD	17.62	0.0000
LM_adj_.	21.64	0.0000
$\tilde{∆}$	12.307	0.000
${\tilde{∆}}_{\mathrm{adj}.}$	14.879	0.000

**Table 3:** Results of the CIPS unit root test.

Variables	Constant	Constant + Trend
CO	−2.061	−2.102
D(CO)	−4.328 ***	−4.269 ***
EF	−1.823	−2.012
D(EF)	−3.976 ***	−4.124 ***
GOV	−1.905	−2.201
D(GOV)	−3.863 ***	−4.007 ***
ITF	−2.076	−1.975
D(ITF)	−3.561 ***	−3.967 ***
EDU	−1.972	−2.475
D(EDU)	−3.937 ***	−4.033 ***

*** significant at 1% significance level.

**Table 4:** Results of the bootstrap Granger causality test between CO and EF.

Country	EF ↛ CO		CO ↛ EF	
	Wald Statistic	*p* Value	Wald Statistic	*p* Value
Belgium	1.904	0.592	6.166	0.104
Bulgaria	0.134	0.714	0.033	0.857
Croatia	0.548	0.459	0.539	0.463
Cyprus	1.497	0.473	11.812	0.003
Czechia	5.264	0.022	0.078	0.780
Denmark	11.336	0.010	6.331	0.097
Estonia	2.110	0.550	0.758	0.859
Finland	0.301	0.860	5.814	0.055
France	0.243	0.886	2.332	0.312
Greece	2.989	0.393	7.974	0.047
Hungary	2.661	0.447	2.847	0.416
Ireland	7.039	0.030	1.467	0.480
Italy	0.918	0.338	0.146	0.703
Latvia	0.241	0.623	0.024	0.878
Lithuania	0.569	0.451	0.314	0.575
Malta	0.114	0.735	0.233	0.629
Netherlands	0.050	0.822	0.038	0.846
Poland	0.527	0.768	1.177	0.555
Portugal	11.864	0.008	11.403	0.010
Romania	4.812	0.090	1.419	0.492
Slovakia	0.232	0.630	0.404	0.525
Slovenia	0.276	0.600	0.088	0.767
Spain	17.824	0.000	2.096	0.553
Sweden	0.188	0.665	0.119	0.730
Panel	72.685	0.012	59.998	0.115

Note: Optimal lag length is selected considering the Schwarz information criterion and bootstrap probability values are produced from 10,000 replications.

**Table 5:** Results of the bootstrap Granger causality test between CO and GOV.

Country	GOV ↛ CO		CO ↛ GOV	
	Wald Statistic	*p* Value	Wald Statistic	*p* Value
Belgium	11.123	0.011	4.295	0.231
Bulgaria	4.301	0.231	14.027	0.003
Croatia	0.434	0.510	0.357	0.550
Cyprus	17.826	0.000	1.534	0.674
Czechia	14.488	0.000	1.262	0.261
Denmark	1.957	0.581	1.435	0.697
Estonia	0.015	0.902	3.204	0.073
Finland	1.105	0.293	2.373	0.123
France	1.173	0.279	0.809	0.368
Greece	5.129	0.163	39.772	0.000
Hungary	1.992	0.369	0.393	0.822
Ireland	10.915	0.012	3.893	0.273
Italy	4.077	0.043	2.992	0.084
Latvia	0.372	0.542	0.043	0.837
Lithuania	0.010	0.919	0.616	0.432
Malta	19.505	0.000	11.111	0.011
Netherlands	0.560	0.454	0.027	0.870
Poland	0.738	0.390	0.024	0.877
Portugal	1.576	0.209	0.094	0.760
Romania	0.092	0.761	2.601	0.107
Slovakia	0.394	0.530	0.077	0.781
Slovenia	7.130	0.068	0.668	0.881
Spain	8.221	0.016	12.902	0.002
Sweden	4.949	0.084	3.019	0.221
Panel	118.558	0.000	109.128	0.000

Note: Optimal lag length is selected considering the Schwarz information criterion and bootstrap probability values are produced from 10,000 replications.

**Table 6:** Results of the bootstrap Granger causality test between CO and ITF.

Country	ITF ↛ CO		CO ↛ ITF	
	Wald Statistic	*p* Value	Wald Statistic	*p* Value
Belgium	5.830	0.120	1.728	0.631
Bulgaria	1.450	0.484	0.886	0.642
Croatia	7.203	0.066	4.554	0.208
Cyprus	0.026	0.873	1.633	0.201
Czechia	1.598	0.206	0.036	0.849
Denmark	0.208	0.976	10.896	0.012
Estonia	3.894	0.273	1.097	0.778
Finland	0.586	0.900	4.599	0.204
France	7.989	0.046	6.689	0.082
Greece	3.675	0.299	0.293	0.961
Hungary	9.157	0.010	0.484	0.785
Ireland	0.372	0.542	0.256	0.613
Italy	5.517	0.019	2.039	0.153
Latvia	0.008	0.928	0.015	0.904
Lithuania	0.077	0.781	0.550	0.458
Malta	0.284	0.594	0.018	0.893
Netherlands	2.390	0.122	0.093	0.760
Poland	0.688	0.709	1.529	0.466
Portugal	1.480	0.224	0.363	0.547
Romania	3.717	0.156	1.524	0.467
Slovakia	0.170	0.680	0.052	0.820
Slovenia	1.555	0.212	0.000	0.986
Spain	32.018	0.000	11.705	0.008
Sweden	3.297	0.069	0.000	0.996
Panel	95.746	0.000	48.027	0.472

Note: Optimal lag length is selected considering the Schwarz information criterion and bootstrap probability values are produced from 10,000 replications.

**Table 7:** Results of the bootstrap Granger causality test between CO and EDU.

Country	EDU ↛ CO		CO ↛EDU	
	Wald Statistic	*p* Value	Wald Statistic	*p* Value
Belgium	3.030	0.387	7.891	0.048
Bulgaria	3.624	0.305	5.177	0.159
Croatia	4.070	0.044	4.196	0.041
Cyprus	0.592	0.744	2.035	0.361
Czechia	2.768	0.429	1.608	0.658
Denmark	2.579	0.461	13.468	0.004
Estonia	0.921	0.337	0.096	0.757
Finland	1.651	0.199	1.284	0.257
France	0.040	0.842	0.614	0.433
Greece	0.170	0.680	0.076	0.783
Hungary	1.654	0.647	5.390	0.145
Ireland	2.145	0.342	8.262	0.016
Italy	12.134	0.007	2.692	0.442
Latvia	3.282	0.350	26.605	0.000
Lithuania	0.775	0.679	1.928	0.381
Malta	1.557	0.459	0.878	0.645
Netherlands	7.485	0.006	2.238	0.135
Poland	8.569	0.036	4.165	0.244
Portugal	8.245	0.016	0.005	0.997
Romania	2.113	0.549	2.294	0.514
Slovakia	1.045	0.593	1.919	0.383
Slovenia	1.097	0.295	1.174	0.279
Spain	6.386	0.094	4.301	0.231
Sweden	16.965	0.001	1.263	0.738
Panel	87.235	0.000	92.087	0.000

Note: Optimal lag length is selected considering the Schwarz information criterion and bootstrap probability values are produced from 10,000 replications.

## Data Availability

The data were obtained from the databases of the World Bank and Fraser Institute through the following links: https://data.worldbank.org/indicator/EN.ATM.CO2E.PC (accessed on 25 February 2022); https://data.worldbank.org/indicator/SE.TER.ENRR (accessed on 25 February 2022); https://www.fraserinstitute.org/economic-freedom/dataset?geozone=world&page=dataset (accessed on 11 March 2022).
